# Renal Surgery

**Published:** 1894-07-07

**Authors:** 


### PROGRESS IN SURGERY.

### RENAL SURGERY.

**Nephrectomy.**—In discussing this operation, Mr. M'Ardle states[1] that the semi-lunar line is the best for reaching the renal vessels, which, he contended, should be double ligatured and cut before attempting to enucleate the organ. He advocated closure of the outer layer of the meso-colon and complete suture of the abdominal wound. The mucous lining of the ureter should be snipped off, and the end rendered aseptic by corrosive sublimate solution. In reference to nephrectomy for malignant tumours of the kidney in infants, Mr. Bland Sutton says,[2] if we take a broad view of sarcomata in children, we find they are variously characterised by peculiar tissues. In some we find a mass of large spindle cells transversely striated so as to resemble on section voluntary muscular fibre. In another form we get cubical epithelium, arranged as tubules or as solid columns, especially in sarcomata growing from glands. In the renal sarcomata of infants these epithelial structures may be very abundant or almost absent. When they are inconspicuous the tumours are prone to contain the striated spindle cells in great abundance. He has come to the conclusion that nothing in the histological characters warrants any diagnosis as to their probable malignancy. He doubts whether it is good policy to remove the capsule of the kidney along with the tumour, and does not see any necessity for bringing the ureter outside. Considering the immediate and remote risks of intervention, he doubts the advisability of attempting their removal.

**Nephro-Lithotomy.**—Dr. Tuffier quotes[3] a case in which hæmorrhage was prevented during the operation by means of pressure on the renal vessels. After the lumbar incision the kidney was dragged to the surface and its convex border fully exposed. Forceps applied to the pedicle of the kidney produced complete occlusion of the vessels, and an incision was made along the convexity of the kidney and three calculi removed. The kidney wound was subsequenty closed by means of catgut sutures, the forceps were removed from the pedicle, and the external wound sutured. Dr. Tuffier has special forceps for thus compressing the renal vessels, but sometimes uses the fingers of an assistant where the forceps cannot be applied. The kidney must be laid completely bare, as this allows the forceps to be passed beneath the inferior extremity of the organ.

Dr. Brown reports[4] a case in which he found tubercle bacilli in a calculus—apparently phosphatic. The patient was known to have tubercular disease of the urinary tract, but although the urine had often been examined no tubercle bacilli had ever been found. Finally about two or three hundred minute soft calculi were passed, and on crushing one of these and mounting it in blood serum tubercle bacilli were found in large numbers.

Hurry Fenwick reminds[5] us that the symptoms caused by renal calculi, and the destruction they are able to produce, depends upon their situation. If they are loose in the pelvis of the kidney they irritate and inflame the mucous membrane, whilst their weight will cause them from time to time to fall upon the mouth of the ureter and produce backward pressure changes. On the other hand, if they are fixed in a deep calyx or embedded in the tolerant renal substance, they are much less likely to cause mischief. The chief difference in these cases is in the character of the pain. In the embedded stone there is constant and often acute pain located in the kidney, rarely radiating unless it is very severe, and then to the testis of the corresponding side. Colics are generally absent. In the loose pelvic stones we encounter the typical renal colics with the radiation to the shoulder and the leg. Fenwick believes, too, that the urine has a higher specific gravity in the embedded stone than in the loose pelvic stone. Probably the backward renal pressure exerted by the ureteral obstruction would account for this difference. Cortical stones are usually composed of oxalate of lime, while pelvic stones are more often uratic or urato-phosphatic. The loose pelvic stones are much more dangerous than the imbedded cortical calculi. The prognosis of operation is largely dependent upon the presence of suppuration—this being more likely to occur in the loose pelvic stones. Of 42 cases of nephrolithotomy undertaken when there was no suppurative change, collected by Dr. Newman, there was no death; whereas when suppuration was co-existent the death-rate was 43·3 per cent. Cortical stones afford capital results, but the operative result of loose pelvic stones depends a great deal upon the condition of the kidney.

**The Urine after Nephrectomy.**—Max Foshay has noted[6] the changes in two cases. In both cases the maximum daily average of quantity excreted occurred in the third week. The maximum daily amount of solids was reached in the fourth week of the first case and third week of the second. The maximum of urea was found in the third week of one case and the second week of the other. In each case the urea reached its maximum one week before the total solids attained a maximum—an interesting fact but difficult of explanation.

**Floating Kidneys.**—Le Gendre points out[7] as etiological factors in the pathogenesis of floating kidney: (1) It exists almost always in females; (2) it is exceptional in children; (3) it rarely shows itself in young girls before adolescence; (4) it is most frequent in females who have been pregnant; (5) it frequently coincides with atonic gastro-intestinal dyspepsia and dilatation of the stomach; (6) it exists most frequently associated with lax abdominal walls, due to any cause. The corset probably has something to do with its frequent occurrence in women, and he recommends that children who present symptoms of nervousness and atony of the gastro-intestinal canal should be made to wear specially-constructed corsets which will not constrict the base of the thorax.

[1] Lancet, March 10, 1894, p. 612. [2] Medical Week, Feb. 10, 1894, p. 77. [3] Medical Week., Feb. 2, 1894, p. 58. [4] Med. Rec., New York, Jan. 17, 1894. [5] Clinical Journ., Jan. 31, 1894, p. 212. [6] Internat. Med. Mag., Nov., 1893. [7] Med. Record, New York, Feb. 3, 1894, p. 149.

